# Profiling the Research Landscape on Cognitive Aging: A Bibliometric Analysis and Network Visualization

**DOI:** 10.3389/fnagi.2022.876159

**Published:** 2022-04-27

**Authors:** Zahiruddin Othman, Ahmad Shahril Abdul Halim, Khairunnuur Fairuz Azman, Asma Hayati Ahmad, Rahimah Zakaria, Kuttulebbai Nainamohamed Salam Sirajudeen, Adi Wijaya, Aidi Ahmi

**Affiliations:** ^1^School of Medical Sciences, Universiti Sains Malaysia, Kota Bharu, Malaysia; ^2^Department of Basic Medical Sciences, Kulliyyah of Medicine, International Islamic University Malaysia, Kuantan, Malaysia; ^3^Department of Health Information Management, Universitas Indonesia Maju, Jakarta, Indonesia; ^4^Tunku Puteri Intan Safinaz School of Accountancy, Universiti Utara Malaysia, Sintok, Malaysia

**Keywords:** cognition, aging, Harzing’s publish or perish, BibliometriX R, VOSviewer, enhanced strategic diagram

## Abstract

**Objectives:**

This study aimed to profile the cognitive aging research landscape from 1956 to 2021.

**Methods:**

A total of 3,779 documents were retrieved from the Scopus database for the bibliometric analysis and network visualization. By comparing each keyword’s overall connection strength (centrality), frequency (density), and average year of publication (novelty) to the calculated median values acquired from the overlay view of the VOSviewer map, the enhanced strategic diagrams (ESDs) were constructed.

**Results:**

The findings showed an increasing trend in the number of publications. The United States leads the contributing countries in cognitive aging research. The scientific productivity pattern obeyed Lotka’s law. The most productive researcher was Deary, I. J., with the highest number of publications. The collaborative index showed an increasing trend from 1980 onwards. Frontiers in Aging Neuroscience is the most prestigious journal in the field of cognitive aging research. In Bradford core journals zone 1, the top 10 core journals of cognitive aging research provided more than half of the total articles (697, or 55.36 percent).

**Conclusions:**

For the next decades, the trending topics in cognitive aging research include neuropsychological assessment, functional connectivity, human immunodeficiency virus (HIV), decision-making, gender, compensation, default mode network, learning and memory, brain-derived neurotrophic factor (BDNF), obesity, D-galactose, epigenetics, frailty, mortality, mini-mental state examination (MMSE), anxiety, and gait speed.

## Background

The scientific literature has long demonstrated cognitive change as a natural part of aging. The dynamic and variable longitudinal changes in cognitive function that occur inherently during the aging process are referred to as cognitive aging (Harada et al., 2013). However, those who maintain their cognitive function at high levels, even with advancing age, are categorized as successful cognitive aging (Daffner, 2010).

The cascade model of cognitive aging suggested by Birren and Cunningham (1985) emphasized a life course approach to cognitive aging and cognitive performance. According to the model, primary aging is characterized by a steady deterioration in mental function, which is often accompanied by problems with memory (particularly new learning and retention), information processing, language, and other cognitive skills. Secondary aging refers to a loss of fluid and crystallized cognitive capacities caused by a disease process such as dementia, whereas tertiary aging refers to impairments in cognitive function caused by total biological devitalization of the organism before death (Birren and Cunningham, 1985).

**Figure 1:**
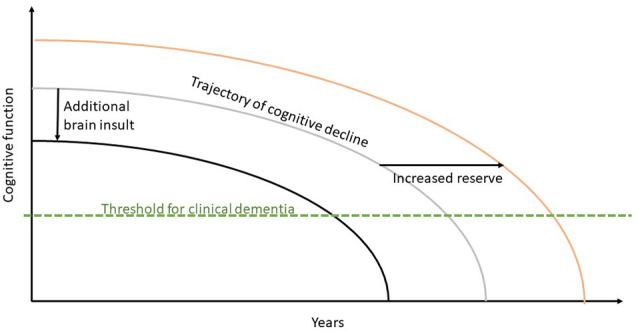
Cognitive decline model. Figure adapted from Krivanek et al. (2021); copyright (2021) with permission from IOS Press.

Brayne and Calloway (1988), on the other hand, depict cognitive decline as a continuum, ranging from normal and successful aging to moderate cognitive impairment and dementia. This model depicts a general downward trend in all elements of cognitive ability, regardless of the competing danger of neuropathological alterations like dementia. However, Krivanek et al. (2021) suggested a new model of cognitive decline that depicts the progression of cognitive decline. In people with neurodegenerative diseases, this theoretical curve would shift to the left. On the other hand, boosting cognitive or brain reserve would shift this theoretical curve to the right, allowing patients to reach this threshold later in life (Figure 1).

Earlier neurocognitive models, which blend behavioral and neurological evidence to develop a conceptual model of cognitive aging, are based on neural compensation. These models, such as the Hemispheric Asymmetry Reduction in Older Adults (HAROLD; Cabeza, 2002), the Compensation-Related Utilization of Neural Circuits Hypothesis (CRUNCH, Reuter-Lorenz and Cappell, 2008), the Posterior-Anterior Shift in Aging (PASA; Davis et al., 2008), and the Scaffolding Theory of Aging And Cognition (STAC and STAC-r; Park and Reuter-Lorenz, 2009; Reuter-Lorenz and Park, 2014), postulate that older adults can perform as well as young adults on cognitive tasks depending on their capacity to recruit additional neural networks. These models, however, were unable to fully explain the cognitive deterioration that happens with healthy aging. Ebaid and Crewther (2020) then offered a theory of cognitive aging based on a system biology approach that combines the sensory deprivation hypothesis, the information degradation hypothesis, and the common-cause hypothesis. The theory stressed the significance of including all of the biological changes that frequently occur at a later age (Ebaid and Crewther, 2020).

Many studies on cognitive aging including its theories have been conducted in the past but only a few have kept track of the literature. The impact of literature on future research could be determined by bibliometric analysis, which is a quantitative analysis of publication metadata. The application of bibliometric approaches in the scientific and professional community has progressed much beyond the basic concept of simple lists of scientific production or citation indexing, and there is a wide range of applications across disciplines (Ellegaard, 2018). This is owing to advancements in bibliometric software such as VOSviewer, Gephi, and Leximencer, as well as the availability and accessibility of scientific databases like Web of Science and Scopus (Donthu et al., 2021).

Previous bibliometric studies have investigated general aspects of aging, namely, aging or oldest age or geriatric (Lund and Wang, 2020; Gonzalez-Alcaide et al., 2021), healthy aging (Gu et al., 2019), aging in combination with other issues such as reception by the scientific community (Glänzel and Schoepflin, 1995), physical therapy (Arnal-GÓmez et al., 2020), geriatric nursing (Ghamgosar et al., 2021), mobile technologies (Tajudeen et al., 2022), safety in-home care (Cao et al., 2021), subjective well-being (Dominko and Verbič, 2019), and specific to aging policies in China (Nan et al., 2020). This bibliometric analysis and network visualization, on the other hand, was carried out to explore the literature on cognitive aging in the Scopus database. It aimed to answer the following research questions:

1. How far has cognitive aging research progressed in terms of publication?
2. What is the scientific productivity pattern in the field of cognitive aging research?
3. Who are the most productive authors in the field of cognitive aging research?
4. What is the present state of collaboration in the field of cognitive aging research?
5. What is the pattern of research on cognitive aging that is scattered?
6. What are the main areas of cognitive aging research?

Therefore, in the present study, we attempted to reveal the publishing trends, scientific productivity patterns, the most productive authors, collaboration status, research patterns across the sources, and the major areas of cognitive aging research.

## Methods

### Data Collection

**Figure 2:**
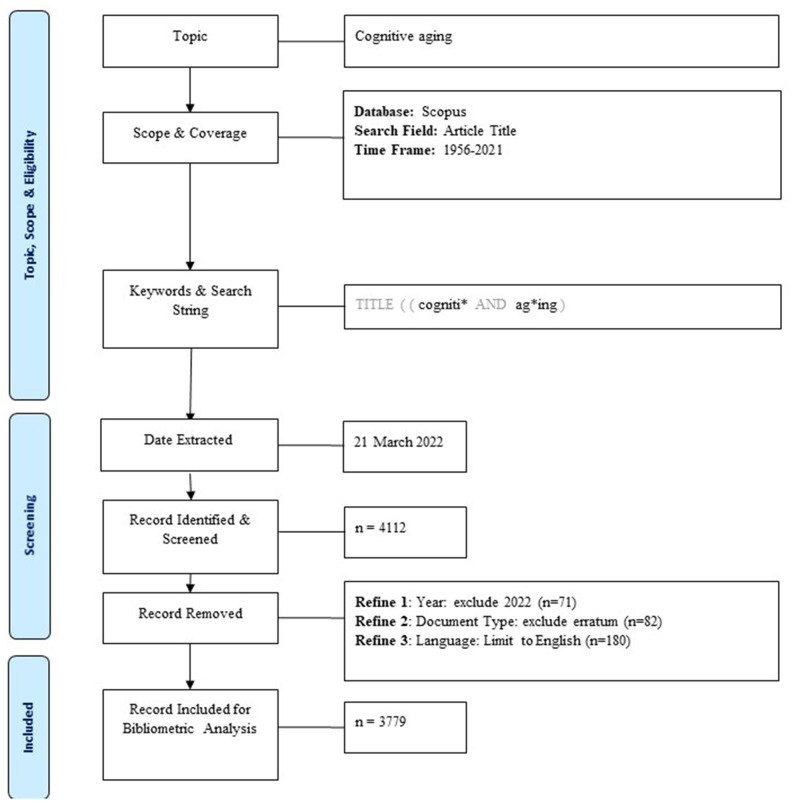
PRISMA flow chart of data inclusion and exclusion (Page et al., 2021).

This is a bibliometric study, which is a computer-assisted review procedure for identifying core research or authors, as well as their relationships by examining all publications related to a specific topic or field (De Bellis, 2009). The data for this study were retrieved and downloaded from the Scopus database on March 21, 2022. From 1956 to 2021, the search term “cogniti* AND ag*ing” in the article title was utilized. We included all the documents written in English from 1956 to 2021. We excluded the 2022 documents (*n* = 71), since the 2022 data is incomplete, and the erratum (*n* = 82) to avoid double counting. Finally, 3,779 documents were identified and downloaded for further analysis (Figure 2).

### Data Analysis

We have combined performance analysis and network analysis to answer our research objectives. The performance analysis, which includes citation- and publication-related metrics, was conducted using Harzing’s Publish or Perish (Harzing, 2007) and BibliometriX R package software (Aria and Cuccurullo, 2017). The author’s keywords were mapped using VOSviewer software (version 1.6.17), a popular tool with a simple graphic interface that can be used to create an author’s keyword co-occurrence map (Van Eck and Waltman, 2021). It allows for identifying significant research subjects and finding large research clusters related to cognitive aging.

Based on a study by Feng et al. (2021), we have created the improved enhanced strategic diagram (ESDs) with the x-axis representing centrality, the y-axis representing density, and the z-axis representing time on a three-dimensional plane. Centrality is a metric that measures the degree of interaction between networks (Cobo et al., 2011; Feng et al., 2021). A theme with a higher centrality score has more external connections to other themes (external strength) and hence has a bigger impact on the development and evolution of the research field (Cobo et al., 2011; Hansen et al., 2020; Feng et al., 2021). This study used the mean strength value of external links to other subjects, i.e., total link strength (TLS), to determine centrality. TLS values equal to or more than the calculated median value were regarded as high centrality, while those less than the median value were regarded as poor centrality.

The density of a topic, on the other hand, is used to determine the topic’s internal strength or degree of interaction within a network (Cobo et al., 2011; Feng et al., 2021). The density of the author’s keywords was determined in this study using co-occurrences. The median value was computed, and co-occurrence values equal to or higher than it was regarded as high density, while those below it were regarded as low density. The novelty of the study, on the other hand, is reflected by time (Feng et al., 2021), and the average publication year was employed in this study. In terms of novelty, the median value of the average publication year was determined, and average publication years equal to or greater than the median were regarded as novel and* vice versa*.

## Results

This study analyzed the main bibliometric indicators to profile the research landscape on cognitive aging from 1956 to 2021.

### Publication and Citation Trend

**Figure 3:**
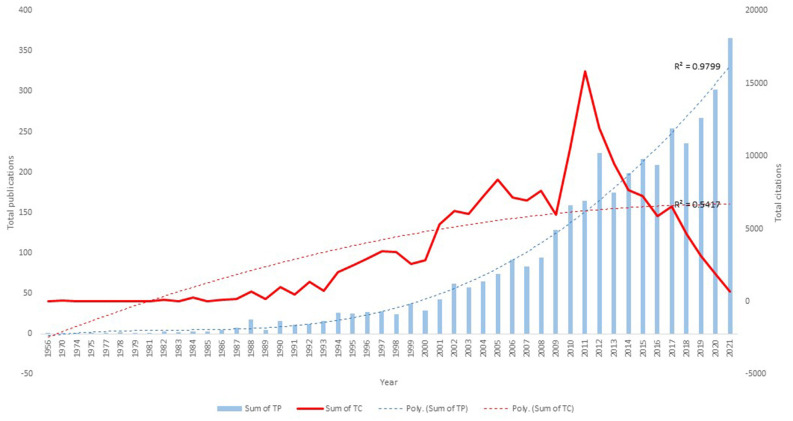
Total publications and citations on cognitive aging from 1956 to 2021.

There were 3,779 publications on cognitive aging retrieved from the Scopus database for this study. The first publication, “The judgment of ambiguous stimuli as an index of cognitive functioning in aging”, was included in the analysis (Basowitz and Korchin, 1956). The number of publications related to cognitive aging remained in the single digits every year until 1987. The publication has been steadily expanding since then (Figure 3). Over the previous three decades, a rapid increase in publications has been reported (1991–2000: 236 or 6.22 percent; 2001–2010: 858 or 22.71 percent; 2011–2020: 2,247 or 59.48 percent). The trend line shows that the number of publications increases polynomially (*R*^2^ = 0.9799), which is greater than a linear increase. In terms of citations, the overall number of citations per year showed a steady increase and an inverse correlation after 2011. The trend line shows that total citations increase polynomially (*R*^2^ = 0.5417), indicating that citations are on an increasing trend, although in the last decade the increase has not been as high as in previous decades.

### Geographical Distribution of the Publications

Table 1 shows the most productive countries based on the number of publications. The United States was the most prolific country and contributed almost half of the total publications. This was followed by the United Kingdom (*n* = 472 or 12.49%), Canada (*n* = 303 or 8.02%), Germany (*n* = 238 or 6.30%) and Australia (*n* = 219 or 5.80%). In terms of total citations, the United States had the lead in citations, followed by the United Kingdom, Canada, and Germany. However, France (10,963) surpassed Australia (8,432), Italy (5,062), and China (4,608).

**Table 1:** Top 10 countries contributed to publications on cognitive aging.

Country	TP	TC	NCP	C/P	C/CP	*h*	*g*
United States	1,769	104,804	1,644	59.24	63.75	157	263
United Kingdom	472	24,650	435	52.22	56.67	72	143
Canada	303	18,871	277	62.28	68.13	67	132
Germany	238	11,947	219	50.20	54.55	58	103
Australia	219	8,432	205	38.50	41.13	48	83
China	206	4,608	175	22.37	26.33	37	60
Italy	173	5,062	157	29.26	32.24	38	66
France	158	10,963	144	69.39	76.13	37	103
Netherlands	154	7,439	149	48.31	49.93	49	83
Sweden	147	7,013	137	47.71	51.19	44	82

*TP, total publication; TC, total citations; NCP, number of cited articles; C/P, citations per article; C/CP, citations per cited articles; *h*, h-index; *g*, g-index*.

### Scientific Productivity Pattern

A total of 12,928 authors contributed to the publications of cognitive aging research. Table 2 lists the number of publications each author has contributed. The majority of the authors have only published once. Between 1956 and 2021, nearly a quarter of the authors contributed at least two articles on cognitive aging.

**Table 2:** Number of publications on cognitive aging contributed by each author.

Number of publications	Number of authors	Percentage
1	9,880	76.4%
2	1,605	12.4%
3	607	4.7%
4	301	2.3%
5	140	1.1%
6	100	0.8%
7	72	0.6%
8	49	0.4%
9	29	0.2%
10	32	0.2%
>10	113	0.9%
**Total**	**12,928**	**100.00%**

### Most Productive Authors

The top 10 most productive authors are listed in Table 3. Based on the number of publications each had published, Deary, I.J., Petersen, R.C., and Brayne, C. were the three major contributing authors. However, Petersen, R.C. obtained the highest citations in terms of total citations, followed by Deary, I.J. and Brayne, C. The topmost cited article in cognitive aging co-authored by Petersen R.C. (Albert et al., 2011), may have contributed to this finding. H-index (Hirsch, 2005) is a measure of the broad impact of researchers’ scientific achievement, especially in sciences and medicine. All the highly productive authors had a value above 20, except for Brodaty, H. and Sachdev, P.S.

**Table 3:** Top 10 authors contributed to publications on cognitive aging.

Author	TP	TC	NCP	C/P	C/CP	*h*	*g*
Deary, I.J.	66	3,761	64	56.98	58.77	33	61
Petersen, R.C.	53	9,989	48	188.47	208.10	27	53
Brayne, C.	51	3,693	49	72.41	75.37	31	51
Matthews, F.E.	42	3,082	40	73.38	77.05	25	42
Mielke, M.M.	40	1,405	38	35.13	36.97	22	37
Knopman, D.S.	39	2,467	37	63.26	66.68	24	39
Starr, J.M.	39	2,498	38	64.05	65.74	24	39
Brodaty, H.	34	1,198	30	35.24	39.93	18	34
Roberts, R.O.	31	2,295	30	74.03	76.50	23	31
Sachdev, P.S.	30	1,110	27	37.00	41.11	18	30

*TP, total publication; TC, total citations; NCP, number of cited articles; C/P, citations per article; C/CP, citations per cited articles; *h*, h-index; *g*, g-index*.

### Collaboration Status

**Figure 4:**
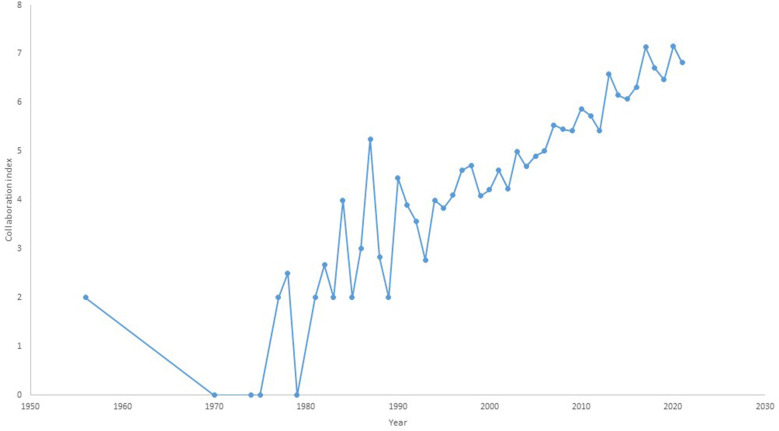
Collaboration index each year from 1956 to 2021 in cognitive aging research.

The collaboration status of cognitive aging research was measured using the collaboration indices as listed in Table 4. Only one published article related to cognitive aging between 1956 and 1965 and the total publications started to increase in the mid-1980s onwards and this could be explained by the progress in cognitive aging research worldwide (Anderson and Craik, 2017). Only 405 or 10.72% were single-authored documents. The majority of the articles published between 1956 and 2021 had multi-authored documents, indicating collaboration. The co-authorship of four researchers on average (mean CI = 4.42) resulted in these multi-authored publications as shown by the collaboration index (Figure 4).

**Table 4:** Collaboration indices.

Year	TP	TNA	SAP	%	MAP	%	TNA_MAP_	CI
1956–1960	1	2	0	0.00	1	100.00	2	2
1961–1965	0	0	0	0.00	0	0.00	0	0
1966–1970	1	1	1	100.00	0	0.00	0	0
1971–1975	2	2	2	100.00	0	0.00	0	0
1976–1980	4	8	1	25.00	3	75.00	7	2.33
1981–1985*	12	27	1	8.33	9	75.00	26	2.89
1986–1990*	52	134	19	36.54	32	61.54	115	3.59
1991–1995*	90	253	20	22.22	64	71.11	233	3.64
1996–2000*	145	526	28	19.31	115	79.31	498	4.33
2001–2005	301	1,226	50	16.61	251	83.39	1,176	4.69
2006–2010*	557	2,650	91	16.34	465	83.48	2,559	5.50
2011–2015*	979	5,326	103	10.52	874	89.27	5,223	5.98
2016–2020*	1,269	8,212	71	5.59	1,196	94.25	8,141	6.81
2021	366	2,391	18	4.92	348	95.08	2,373	6.82

TP, total publications; TNA, total number of authors; SAP, single author publications; MAP, multiple authors publications; TNA_MAP_, total number of authors in MAP; CI, collaboration index = number of authors in the MAP/number of MAP. *1985—2 documents with 0 author; 1987—1 document with 0 author; 1994—2 documents with 0 author; 1995—4 documents with 0 author; 1997—1 document with 0 author; 1999—1 document with 0 author; 2006—1 document with 0 author; 2015—2 documents with 0 author; 2016—1 document with 0 author; 2019—1 document with 0 author.

### Scattering Pattern of Research Work Across the Sources

The distribution of document sources was assessed using Bradford (1950) to establish the scattering pattern of research on cognitive aging. The decreasingly ordered document sources were divided into three zones, each of which had an average number of 1,260 documents. Table 5 shows that there were 32 Bradford’s core journals (Zone 1 or nucleus) with 1,259 articles, Zone 2 had 187 journals (1,275 articles), and Zone 3 had 965 journals (1,245 articles).

**Table 5:** Distribution of the sources and corresponding documents in three zones.

Zone	No. of sources	No. of articles	Percentage
1	32	1,259	33.32
2	187	1,275	33.74
3	965	1,245	32.94
**Total**	**1,184**	**3,779**	**100.00**

Table 6 shows the top 10 core journals of cognitive aging research, which contributed more than half of the total articles (697 or 55.36%) in zone 1. In terms of total citations, Psychology and Aging obtained the highest total citations with two articles ranking among the top 10 most cited (Baltes and Lindenberger, 1997; Bialystok et al., 2004), followed by Neurobiology of Aging, Journals of Gerontology Series B Psychological Sciences and Social Sciences, Frontiers in Aging Neuroscience, and Journal of the American Geriatrics Society. Most of the journals listed in the top 10 core sources were specific to aging research except for PLoS One, which covers many subject areas.

**Table 6:** Top 10 core sources of the research on cognitive aging.

Source title	TP	TC	NCP	C/P	C/CP	*h*	*g*
Frontiers in Aging Neuroscience	111	2,992	105	26.95	28.50	30	50
Neurobiology of Aging	104	5,366	102	51.60	52.61	45	71
Journal of Alzheimer Disease	85	2,238	78	26.33	28.69	27	44
Psychology and Aging	82	7,633	80	93.09	95.41	45	82
Journals of Gerontology Series B Psychological Sciences and Social Sciences	69	3,303	62	47.87	53.27	28	57
Journals of Gerontology Series A Biological Sciences and Medical Sciences	54	2,806	51	51.96	55.02	29	52
Plos One	53	1,902	52	35.89	36.58	25	43
Aging Neuropsychology and Cognition	47	1,095	39	23.30	28.08	16	32
International Journal of Geriatric Psychiatry	46	1,413	44	30.72	32.11	19	37
Journal of the American Geriatrics Society	46	2,888	45	62.78	64.18	27	46

*TP, total publication; TC, total citations; NCP, number of cited articles; C/P, citations per article; C/CP, citations per cited articles; *h*, h-index; *g*, g-index*.

### Main Topics of the Research on Cognitive Aging

**Figure 5:**
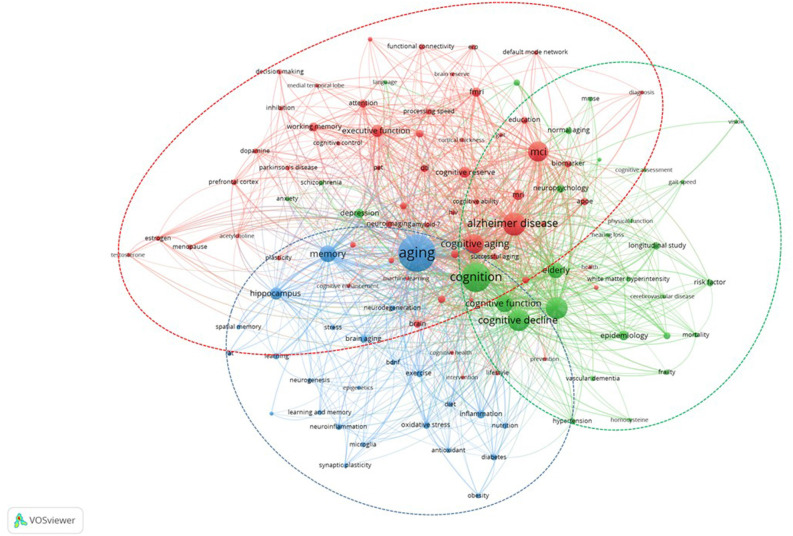
The co-occurrence of the author’s keywords minimum occurrence threshold of 13.

The main topics of cognitive aging research were identified using a keyword co-occurrence analysis. Only 113 of the 5,258 keywords used by the author surpassed the minimum occurrence level of 13 (Figure 5). Analytical (individual-based), descriptive (population-based), and experimental studies are the three clusters that emerge from the map (Table 7).

**Table 7:** The main keywords in each cluster.

Cluster/Focus	Color	Keywords
1/Analytic studies (Individual-based)	Red	Neuroimaging: MRI, fMRI, DTI, PET. Cognitive trajectories: MCI, cognitive aging, healthy aging, successful aging. Cognitive domains: executive function, working memory, attention, episodic memory, processing speed, decision making, intelligence.
2/Descriptive studies (Population-based)	Green	Risk factors: elderly, depression, hypertension, schizophrenia, anxiety. Behavioural assessments: cognitive function, neuropsychology test, frailty, MMSE, language, gait speed, hearing loss, physical function, vision.
3/Experimental studies	Blue	Lifestyle: exercise, nutrition, antioxidant, diet. Animal/human models: diabetes, stress, obesity, D-galactose. Markers: oxidative stress, neuroinflammation, BDNF, microglia, neurogenesis, synaptic plasticity. Hippocampal functions: memory, learning.

*MRI, magnetic resonance imaging; fMRI, functional MRI; DTI, diffusion tensor imaging; PET, positron emission tomography; MCI, mild cognitive impairment; MMSE, mini mental state examination; BDNF, brain-derived neurotrophic factor*.

**Figure 6:**
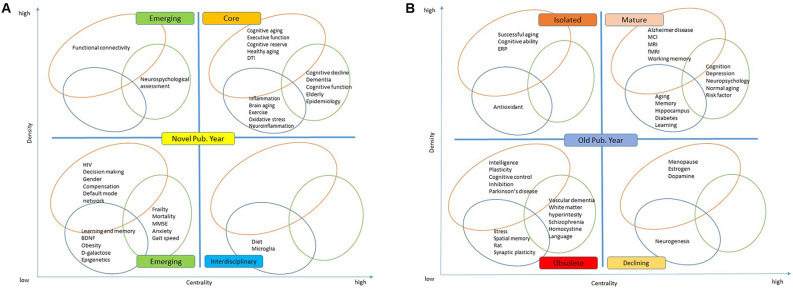
Enhanced strategic diagrams showing **(A)** emerging, core, and interdisciplinary in the novel publication year **(B)** isolated, mature, obsolete, and declining topics in the old publication year.

The ESDs were created by comparing each keyword’s overall link strength (centrality), frequency (density), and average year of publication (novelty) to the derived median values obtained from the overlay view of the VOSviewer map. There are four different types of themes that can be determined based on the plane’s position (Cobo et al., 2011; Feng et al., 2021). The four themes in the novel publication year are depicted in Figure 6A. Emerging with high density (upper-left quadrant), emerging with low density (lower-left quadrant), core (upper-right quadrant), and interdisciplinary (lower-right quadrant) are the four categories (lower-right quadrant). Figure 6B depicts the four themes that existed in the old publication year: isolated (upper-left quadrant), obsolete (lower-left quadrant), mature (upper-right quadrant), and declining (lower-right quadrant).

## Discussion

The cognitive aging theory was first introduced by Welford and Birren in 1965 (Birren, 1965). Before 1965, cognitive aging research was descriptive, determining which areas of intellectual performance are impaired in older vs. younger persons (Anderson and Craik, 2017). Over the past three decades, the growing number of publications meant that research on cognitive aging was gaining traction around the world. This was in line with the findings on healthy aging and geriatric nursing research reported by Gu et al. (2019) and Ghamgosar et al. (2021), respectively. Furthermore, this continual increase in research has important clinical and intellectual implications, as it aids clinicians in better measuring, preventing, and treating cognitive aging by establishing individualized risk profiles connected to a personalized intervention strategy (Ryan et al., 2019). The total citations peaked in 2011, suggesting this to be the key year for the development of the field (Cao et al., 2021). Further investigation revealed that the National Institute on Aging-Association Alzheimer’s working groups on Alzheimer’s disease diagnostic criteria issued a set of recommendations for diagnosing mild cognitive impairment attributable to Alzheimer’s disease in 2011 (Albert et al., 2011).

The United States was the most prolific country followed by the United Kingdom, Canada, Germany, and Australia. The productivity of the top five countries was in line with the recent study conducted by Arnal-GÓmez et al. (2020). The authors suggested that productivity was related to the aging of their population, as shown by the positive correlation between productivity and the aged population. However, in terms of scientific productivity pattern, as the number of publications contributed increased, the number of authors declined. This was consistent with Lotka’s Law (Lotka, 1926), which stated that single-publication authors are far more likely to conduct subsequent research on similar research areas (Rowlands, 2005; Kushairi and Ahmi, 2021).

The collaboration index was on the rise, reflecting the growing complexity of multidisciplinary research and the increasing quantity and quality of the resultant publications in cognitive aging (Stallings et al., 2013). According to several studies, research conducted by larger groups is more influential and impactful (Crane, 1972; Goffman and Warren, 1980). Furthermore, articles co-authored by international collaborators receive more citations than articles co-authored by domestic collaborators, implying that internationally co-authored articles represent a larger segment of global science (Narin et al., 1991). Dutt and Nikam (2015), however, reported that publications from certain prolific countries and institutions emerging from domestic collaboration resulted in a higher impact than those from international collaboration.

Based on the keyword co-occurrence analysis, there are three main clusters. In the analytical (individual-based) studies cluster, the main keywords were grouped into: (i) neuroimaging which includes various modalities; (ii) cognitive trajectories that are related to the major study groups; and (iii) cognitive domains that are related to the cognitive assessment frequently used (Table 7). The mature keywords are Alzheimer disease, mild cognitive impairment (MCI), magnetic resonance imaging (MRI), functional MRI (fMRI), and working memory, while the core keywords include cognitive aging, executive function, cognitive reserve, healthy aging, and diffusion tensor imaging (DTI). These keywords highlight the main group of individuals, established neuroimaging, and cognitive domains related to analytical (individual-based) studies.

Individuals in the aging population vary greatly, and while some develop cognitive impairment (including mild cognitive impairment), Alzheimer’s disease, and other types of dementia, others may retain their cognitive function to a major extent well into old age, which is also known as healthy aging (Nyberg et al., 2012). Reduced brain volume, cortical thinning, and deterioration in white matter microstructure are common age-related structural alterations (Fjell and Walhovd, 2010), which can contribute to lower cognitive performance in domains like executive function, memory, and processing speed (Nyberg et al., 2012; Grady et al., 2016; Cabeza et al., 2018). The cognitive reserve theory attempts to explain why some people can sustain cognitive performance while having a disease or aging-related brain abnormalities. Individuals with a larger cognitive reserve are thought to process information more efficiently, allowing them to functionally adapt to brain aging and sustain greater disease before cognitive deficits appear (Stern, 2002).

While the emerging keywords include functional connectivity, human immunodeficiency virus (HIV), decision making, gender, compensation, and default mode network, it is generally known that functional connectivity alterations associated with Alzheimer’s disease start years before structural changes and clinical symptoms are noticed (Cieri and Esposito, 2018). In persons at risk of developing Alzheimer’s disease, some resting-state fMRI studies have found increased functional connectivity between certain regions of the default network, while others have found decreased connectivity (Cieri and Esposito, 2018). Overactivation in functional connectivity across resting-state networks may be related to compensatory mechanisms even in cognitively preserved older adults, according to some studies (Li et al., 2015; Grady et al., 2016; Fjell et al., 2017). More advanced neuroimaging techniques with a higher spatial-temporal resolution, as well as methods to measure neurotransmitter activity or gene expression in real-time, may be developed, allowing for a better knowledge of the brain factors associated with cognitive aging and a new avenue for intervention (Anderson and Craik, 2017).

With a higher number of older individuals living with HIV in the era of antiretroviral therapy, there is a higher likelihood of cognitive decline, particularly in executive function, processing speed, vocabulary, recollection, and motor/psychomotor domains (Deng et al., 2021). In the available research, there is some evidence for premature and accelerated cognitive aging among HIV individuals, particularly in large and longitudinal studies and those with a higher number of older samples. Future HIV and cognitive aging studies will need to standardize neuropsychological testing methodologies and outcomes, as well as use a large sample from collaborative multi-centers (Aung et al., 2021).

Decision-making deficit has been shown even in cognitively healthy older adults (Spreng et al., 2016; Bangma et al., 2017). It may increase vulnerability to fraud (Duke Han et al., 2016; Lamar et al., 2020), including financial exploitation. Weissberger et al. (2020) found that perceived financial exploitation in old age is linked to differences in whole-brain functional connectivity involving the hippocampus, insula, and medial frontal cortex, which is consistent with models linking age-related changes in decision-making and social cognition to financial exploitation.

There is still debate on the gender difference in cognitive performance with a particular interest in the older population. Previous studies have indicated that sex variations in cognitive performance remain until late adulthood (Siedlecki et al., 2019), as well as an unbalanced prevalence of neurodegenerative diseases associated with different cognitive impairments, for example, males are more likely to suffer from MCI and Parkinson’s disease, while females are more likely to suffer from Alzheimer’s disease (Cholerton et al., 2018; Sohn et al., 2018). Different interrelationships between cognitive functions could potentially explain sections of these different age-related trajectories, presenting a promising study topic.

Large-scale functional brain networks have also been used to investigate neurocognitive aging (Damoiseaux, 2017). Internally directed cognitive processes that rely on access to prior-knowledge representations to guide goal-directed behaviors generally engage the default network (Andrews-Hanna et al., 2014). During the performance of externally directed tasks, however, default-network are suppressed (Buckner et al., 2008). Reduced suppression, decreased within-network connectivity, and increased between-network connectivity are all age-related alterations in the default network, all of which are minimally controlled by task context (Spreng and Schacter, 2012; Rieck et al., 2017). These led to a default-executive coupling hypothesis of aging proposed by Spreng and Turner (2019). This hypothesis was based on findings that the lateral prefrontal cortex, responsible for executive function and cognitive control, is functionally coupled with engagement of the default network in old age.

In the descriptive (population-based) studies cluster, the main keywords were grouped into: (i) risk factors; and (ii) behavioral assessment. The mature keywords in this cluster include cognition, depression, neuropsychology, normal aging, and risk factor, while the core keywords were cognitive decline, dementia, cognitive function, elderly, and epidemiology. These keywords highlight the common types of population-based studies (epidemiology and longitudinal), established risk factors (elderly, depression, etc.), and behavioral assessments (cognitive function, frailty, mini-mental state examination (MMSE), etc.).

The emerging keywords in this cluster were neuropsychological assessment, frailty, mortality, MMSE, anxiety, and gait speed. These keywords reflect different behavioral assessments, frailty, gait speed, and MMSE, frequently used in population-based studies. The International Academy on Nutrition and Aging and the International Association of Gerontology and Geriatrics defined cognitive frailty as comorbid physical frailty (>1 Fried criteria) and mild cognitive impairment (Petersen criteria; Kelaiditi et al., 2013; Rivan et al., 2020). Physical frailty such as gait speed and handgrip strength has been linked to cognitive decline in older persons in many previous studies (Demnitz et al., 2016; Kobayashi-Cuya et al., 2018; Chou et al., 2019). Furthermore, a recent theory has suggested a link between cognitive impairment, sensory deprivation, and common-cause hypotheses (Ebaid and Crewther, 2020).

In the experimental studies cluster, the main keywords were grouped into: lifestyle, animal/human models, cognitive markers, and hippocampal functions. The mature keywords in this cluster include aging, memory, hippocampus, diabetes, and learning, while the core keywords were inflammation, brain aging, exercise, oxidative stress, and neuroinflammation. This type of study normally assesses the role of lifestyle in affecting cognitive markers as well as hippocampal functions in animals and humans (Fordyce and Wehner, 1993; Vaynman et al., 2004; Gow et al., 2012; Woodard et al., 2012). The Cam-CAN data set provides a valuable resource that contributes to the expanding understanding of cognitive aging as a lifetime developmental process characterized by intricate interactions across life stages and cognitive domains. Thus, there is a need for large-scale cognitive aging experimental studies to include a wider range of ages and cognitive tasks (Shafto et al., 2020).

Learning and memory, brain-derived neurotrophic factor (BDNF), obesity, D-galactose, and epigenetics were among the emerging keywords in the experimental studies cluster. BDNF, a protein that regulates synaptic transmission and induces long-term changes in excitability and synaptic plasticity in the adult brain, has been shown to have a prominent role in neuron survival, growth, and function in experimental models (Miranda et al., 2019). The BDNF Val66Met polymorphism, which regulates BDNF expression, has been linked to resilience toward the effects of aging on cognition (Collins et al., 2021). In addition, epigenetics has been studied as a possible relationship between environmental/lifestyle factors (hormone status, food, stress, and exercise) and the variability of cognitive function as people age (Barter and Foster, 2018; Beydoun et al., 2020).

With a few exceptions, we have addressed all of the research objectives. To begin with, we only searched one database, Scopus, because it is the most comprehensive database (Zhu and Liu, 2020; Pranckute, 2021) and to avoid variations in data formats and field tags that would occur if we used data from multiple databases. Second, in order to prevent finding unnecessary documents, we run our search in the article title; nonetheless, we may overlook certain significant documents. Third, keyword cleaning and statistics are tailored to our specific needs, which may be limited by our professional knowledge and experience.

## Conclusions

The United States continues to dominate in terms of publication and research collaboration in cognitive aging. The journals publishing themes relevant to aging research are the top sources of cognitive aging research. In the coming decades, the hot topics in cognitive aging research would be neuropsychological assessment, functional connectivity, HIV, decision-making, gender, compensation, default mode network, learning and memory, BDNF, obesity, D-galactose, epigenetics, frailty, mortality, MMSE, anxiety, and gait speed. These study findings provide useful references to health practitioners and researchers who are involved in cognitive aging management.

## Author Contributions

ZO and ASAH planned the study. RZ, KFA, and KNSS collected the data and drafted the manuscript. AHA, AW, and AA revised the manuscript and language. All authors contributed to the article and approved the submitted version.

## Conflict of Interest

The authors declare that the research was conducted in the absence of any commercial or financial relationships that could be construed as a potential conflict of interest.

## Publisher’s Note

All claims expressed in this article are solely those of the authors and do not necessarily represent those of their affiliated organizations, or those of the publisher, the editors and the reviewers. Any product that may be evaluated in this article, or claim that may be made by its manufacturer, is not guaranteed or endorsed by the publisher.
